# High-content method for mechanosignaling studies using *IsoStretcher* technology and quantitative Ca^2+^ imaging applied to Piezo1 in cardiac HL-1 cells

**DOI:** 10.1007/s00018-024-05159-6

**Published:** 2024-03-14

**Authors:** Anna-Lena Merten, Ulrike Schöler, Yang Guo, Fabian Linsenmeier, Boris Martinac, Oliver Friedrich, Sebastian Schürmann

**Affiliations:** 1 Institute of Medical Biotechnology, Department of Chemical and Biological Engineering, Friedrich-Alexander-Universität Erlangen-Nürnberg (FAU), Paul-Gordan-Str. 3, 91052 Erlangen, Germany; 2 School in Advanced Optical Technologies, Friedrich-Alexander-Universität Erlangen-Nürnberg, Paul-Gordan-Str. 6, 91052 Erlangen, Germany; 3 Victor Chang Cardiac Research Institute, 405 Liverpool St, Darlinghurst, NSW 2010 Australia; 4 School of Clinical Medicine, St Vincent’s Healthcare Clinical Campus, University of New South Wales, Darlinghurst, NSW 2010 Australia

**Keywords:** Mechanobiology, Mechanotransduction, Mechanosensitive, Cell stretching, Cardiomyocytes, GsMTx4, Yoda1

## Abstract

The importance of mechanosensory transduction pathways in cellular signalling has prominently come to focus in the last decade with the discovery of the Piezo ion channel family. Mechanosignaling involving Piezo1 ion channels in the function of the heart and cardiovascular system has only recently been identified to have implications for cardiovascular physiology and pathophysiology, in particular for heart failure (i.e., hypertrophy or dilative cardiomyopathy). These results have emphasized the need for higher throughput methods to study single-cell cardiovascular mechanobiology with the aim of identifying new targets for therapeutic interventions and stimulating the development of new pharmacological agents. Here, we present a novel method to assess mechanosignaling in adherent cardiac cells (murine HL-1 cell line) using a combination of isotropic cell stretch application and simultaneous Ca^2+^ fluorescence readout with quantitative analysis. The procedure implements our *IsoStretcher* technology in conjunction with a single-cell- and population-based analysis of Ca^2+^ signalling by means of automated image registration, cell segmentation and analysis, followed by automated classification of single-cell responses. The method is particularly valuable for assessing the heterogeneity of populations with distinct cellular responses to mechanical stimulation and provides more user-independent unbiased drug response classifications.

**Supplementary Information:**

The online version contains supplementary material available at 10.1007/s00018-024-05159-6.

## Introduction

The cardiovascular system (CVS) is under the continuous influence of mechanical forces and stresses. When the heart contracts and pumps blood around the body, it also generates strain, pressure and shear forces, all of which are permanently monitored by the CVS as feedback, resulting in several auto-regulatory loops. Examples comprise the Frank-Starling mechanism affecting stroke volume as a function of end-diastolic filling [1] or mechano-electric coupling (MEC) as a beat-to-beat regulation of cardiac rhythm in response to mechanical environment [37]. Such regulatory mechanisms are vital to heart functionality and play an important role in pathological conditions of the heart, such as cardiomyopathy and arrhythmia [25, 39]. Normal cardiac function has been implicated to involve activities of voltage-gated and mechanosensitive ion channels in cardiomyocytes [15], e.g. of the transient receptor potential (TRP) or the Piezo family [49]. Particularly, rather than for their contribution to normal cardiomyocyte function, TRP channels have been shown to be aberrantly activated in disease states and, e.g., contribute to hypertrophic cardiomyopathies [22, 45–47]. For Piezo channels, in contrast, almost nothing is known about their contribution to cardiomyopathies. Therefore, Piezo channels, particularly Piezo1, have very recently gained profound interest within the field of cardiac research [4]. Piezo1 is a non-selective mechanosensitive, i.e.*,* stretch-activated, cation channel [8, 52], and is considered to play a major role in the CVS through its regulation of Ca^2+^ homeostasis [27, 37]. A recently reported study that identified a new Piezo1-controlled signalling pathway in the pathophysiology of cardiac left ventricular hypertrophy (LVH) [59] strongly supports this view.

Methods to study cardiac mechano-responses, at the one end of the spectrum, focus on measuring whole heart electrical activity, while at the other end, they attempt for recordings from single cells or single ion-channels via classical electrophysiology methodologies, e.g., the voltage clamp and patch clamp technique. Both ends of that spectrum are limited by their time-intensive and elaborate methodologies, generating data from very few samples in each time frame only. Across all scales of methodologies to assess cellular and organ mechanosensitivity, there is a demand for higher-throughput methods that allow to examine the activity of mechanosensitive ion channels in their native cellular environment in cell populations and tissue. High-throughput electrophysiology metrologies have made major contributions to allow for ion channel screening and facilitate drug discovery [11, 51], even for native cardiomyocytes [44], however, none of such automated patch-clamp systems so far recreate the complex patterns of applying mechanical stress to each individual cell under investigation.

To overcome those constraints, cell stretching devices have been engineered that can mimic defined mechanical environmental conditions in elastic polymer chambers (typically polydimethylsiloxane (PDMS)) using various stretching mechanisms [14]. In the past, the majority of systems relied on using uniaxial strain, for which most commercial solutions are tuned (reviewed in [14]). Unidirectional strain is, for example, predominant in studies on the interaction between muscles and tendons [34], in which stretch is directly applied to the tendon ends and cells are subjected to strong shear stimuli. Shear also plays an important role in the physiological conditions of cardiovascular endothelial cells and has therefore been studied with various technological approaches [14]. However, given that mechanical stimuli do physiologically act on or within the human body in multiple directions, in recent years, bioengineers have developed multi-axial stretch devices. Biaxial and equi-biaxial distension of tissues seems more the rule rather than the exception, particularly for hollow organs and tissues, such as lung alveoli, gut, bladder, or the heart, creating a large area of need for such metrologies. Some of those devices can also be mounted onto fluorescence microscopes, thus enabling studies of mechanotransduction via simultaneous live-cell imaging during the application of different stretch or stress protocols [13]. To investigate the effect of mechanical forces in hollow organs, such as the heart, there is, therefore, a particular interest in 2D in-plane isotropic stretch mimicking the systolic-diastolic contraction-dilation heart cycle. As a consequence, in particular pneumatically driven elastomer-based systems are not suitable to maintain a steady focal plane for required online live-cell imaging purposes (reviewed in [13]). To close this gap, we have previously developed the *IsoStretcher* system [43] which allows stretch application to 2D substrate-adhered cells or even 3D-hydrogel-embedded cardiomyocytes [13] using custom PDMS stretch chambers of tuneable stiffness. This hardware development must go in hand with adequate process and data management workflows to allow for an automated assessment of mechanotransduction studies of CVS cells in a high-content context. This should also enable the classification of subpopulations of cells that may respond differentially to stretch and/or applied drugs and thus, such an approach is capable of minimising bias that is usually present in low throughput manual user interventions.

**Fig. 1:**
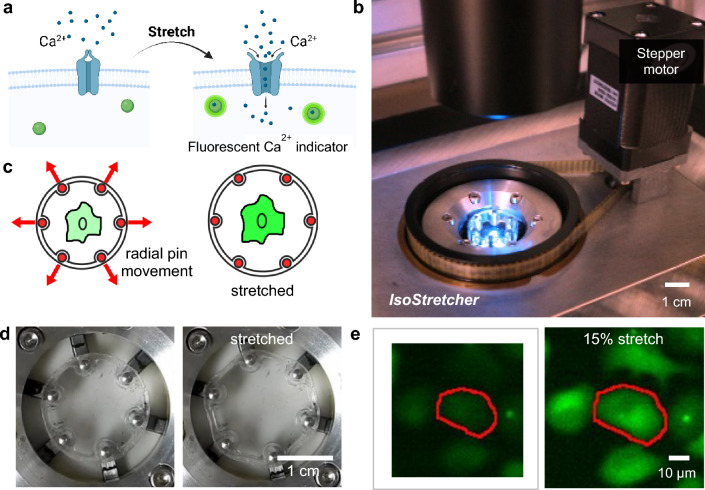
Isotropic cell stretcher technology for functional investigation of mechanosensitive ion channels. **a** Stretch-activated opening of mechanosensitive ion channels, e.g., of the Piezo family, induces cellular Ca^2+^ influx monitored with a fluorescent calcium indicator. **b** *IsoStretcher* system, mounted on a fluorescence microscope, enabling in-plane 2D isotropic stretch. **c** Isotropic stretch of cells adhered to an elastomer membrane is conducted by radial movement of six rigid pins affixed to a custom-made polydimethylsiloxane (PDMS) chamber. **d** Images of the mounted PDMS cell culture chamber at 0% stretch (left) and 15% stretch (right). **e** Fluorescence images of HL-1 cells at 0% stretch (left) and at 15% stretch (right). The increase in cell surface area by stretch, as well as the increase in fluorescence intensity, are clearly visible

In the present study, we introduce a novel method to assess mechanosignaling in cardiac HL-1 cells on a single-cell level with a cell-population-based high-content analysis. For this purpose, we have developed a new version of our *IsoStretcher* system (Fig. 1), featuring major improvements in mechanical precision, and larger cell culture chambers, made with a new custom-designed PDMS casting mould [30]. The technology was applied in isotropic stretch stimulations to cultured HL-1 atrial cardiomyocytes intrinsically expressing mechanosensitive Piezo1 ion channels [21]. The effect of stretch on the cells was determined by preloading the cells with the Ca^2+^-sensitive dye Fluo-4 and recording the global intracellular Ca^2+^ fluorescence during cell stretch using epifluorescence microscopy. We further used Yoda1, a Piezo1 channel agonist [53], to chemically activate/sensitise the channels. For automated analysis of stretch-dependent Ca^2+^ fluorescence on a single cell level, we developed an image processing routine involving image registration to compensate for the stretch motion of the field-of-view (FoV), automated cell segmentation, and subsequent analysis and classification of single-cell data.

Our study confirms stretch-activation of mechanosensitive ion channels as the cause for extracellular Ca^2+^ conveying the increase in the fluorescence intensity upon stretching HL-1 cardiomyocytes [21]. This is also consistent with the findings of recent studies documenting the function of Piezo1 in the healthy and diseased heart [27, 59] and underlines the importance of investigating Piezo1-mediated Ca^2+^ signalling. However, we also observed a heterogeneous behaviour of subpopulations of HL-1 cells, in particular increases in stretch-induced Ca^2+^ levels in HL-1 cells in the presence of the general mechanosensitive channel blocker GsMTx4. To analyse the heterogeneity of mechanotransduction in cell populations, the method presented here should be of value in future studies also involving other cell types.

## Materials and methods

### *IsoStretcher* cell stretching system

To apply isotropic stretch to biological samples and examine their reaction to mechanical stimulation, we previously introduced the *IsoStretcher* [43]. The device and its operating principle are shown in Fig. 1. Briefly, the main principle is to activate mechanosensitive ion channels (Fig. 1a) by stretching cells adhered onto an elastomer bottom membrane within the chamber (Fig. 1c, d) that was casted from PDMS. The *IsoStretcher* device (Fig. 1b) translates a stepper motor-driven rotation into the precise radial motion of six mounting pins.

In detail, a small pulley that is attached to the stepper motor’s shaft connects it to a bigger pulley via a timing belt. That bigger pulley is in turn connected to a rotating cam disk which features six oblique grooves. Six pins hold a PDMS chamber which functions as the flexible seeding substrate for the biological samples, in the centre of the device. Those pins are attached to the front of one slider each, which, in turn, each carries one more pin at the back end. Those pins at the back are inserted into the oblique grooves of the rotating cam disk. Through rotation of the disk, the pins follow the grooves and push or pull the sliders along radial trajectories. Thus, the pins holding the chamber move away from, or towards each other and, therefore, stretch or relax the PDMS chamber, respectively (see Fig. 1d). The technical approach of applying stretch to a sample using the *IsoStretcher* is outlined in Fig. 1c. The chamber, custom-made from PDMS in a casting process, is being pulled along three in-plane axes, which results in a radial stretch of the chamber and of cells adhered to its chamber bottom (representative cell shown in green, not to scale, Fig. 1c). Images of the chamber in an unstretched and stretched state are shown in Fig. 1d.

The chamber can be stretched up to 20% radial increase in steps of 1%, which corresponds to a maximum increase in surface area of 40% [13]. The sample within the chamber can be imaged in real time using an inverted (fluorescence) microscope. This ensures that the reaction of the sample to stimulation can be directly monitored during the experiment.

Compared to the previous version of the *IsoStretcher* [43], the device’s functionality was improved by various hardware and software adjustments to decrease friction (and, therefore, heat production) as well as to enhance the precision of movement [30]. The PDMS chamber design has been revised to allow for bigger samples and more volume (~ 800 µl over previously ~ 100 µl). Furthermore, the chamber geometry has been adjusted to dissipate stresses during the stretch to the wall pillars around the pin holders to keep the optical plane as constant as possible during the stretch. In addition, more durable materials in the moving parts were chosen [30].

### HL-1 cell culture

HL-1 cells were kindly provided by Dr. Felix B. Engel (Nephropathology, Institute of Pathology, University Hospitals Erlangen, FAU). HL-1 cells are derived from the AT-1 mouse atrial cardiomyocyte tumor lineage [7]. They are commonly used as a substitute for primary adult cardiomyocytes as they are unique in showing a differentiated cardiac phenotype with spontaneous contractions while maintaining the ability to continuously divide [58]. The cells were maintained in Claycomb media (Sigma-Aldrich, Steinheim, Germany) supplemented with 10% Fetal Bovine Serum (FBS, Sigma-Aldrich), 1% Penicillin–Streptomycin (10,000 units penicillin and 10 mg streptomycin/ml, Sigma-Aldrich, USA), 1% Norepinephrine (Stock: 10 mM, Sigma-Aldrich), and 1% L-Glutamine (Stock: 200 mM, Sigma-Aldrich), at 37 °C within 5% CO_2_ atmosphere. Trypsin–EDTA (Sigma-Aldrich) was used for splitting and collecting cells. The cell culture flasks used for cultivation were coated with a solution of fibronectin and gelatine (5 µg/ml fibronectin + 0.02% gelatine in water) to promote the adherence of HL-1 cells to the flask bottom. The same coating was applied to the PDMS chambers before seeding cells. For each experiment, one chamber was prepared with 200,000 cells which were incubated at 37 °C for 4 h to allow for adherence. Subsequently, the cells were stained with a fluorescent dye as described below.

### Calcium imaging

HL-1 cells were rinsed with physiological salt solution (PSS) containing 1 mM CaCl_2_ and then incubated with the fluorescent Ca^2+^ indicator Fluo-4 AM (Invitrogen, Darmstadt, Germany) at a concentration of 2 μM in the same PSS mixture, at 37 °C with 5% CO_2_ for 30 min. The cells were maintained in the PSS mixture during the experiment.

Ca^2+^ imaging and data recording were carried out at room temperature on a Nikon Eclipse Ti2-E inverted epifluorescence microscope (Nikon Europe B.V., Düsseldorf, Germany) using a 20 × objective lens (Nikon S Plan Fluor ELWD 20x/0.45) and an Andor Neo sCMOS camera with 2 × 2 binning and 100 ms exposure (10 frames/s). The light source was a xenon lamp (Sutter DG4) connected via a liquid light guide. The total light intensity was measured as 2.0–2.5 mW per field of view (*i.e.* ~ 0.5 mW/mm^2^). This results in an average light intensity of around 0.5 µW per cell, which is very low and not cytotoxic under our experimental conditions [48].

The experiments were divided into four phases of one minute each. In the first phase (P I, pre-stretch), the cells’ idle behaviour was recorded without any mechanical manipulation. P I either contained no additional drug (ctrl.) or cells were pre-incubated with 5 µM of the mechanosensitive channel blocker GsMTx4 (Alomone Labs, Jerusalem, Israel) [17]. At the beginning of the second phase (P II), 5 µM of the Piezo1 channel agonist Yoda1 (Cayman Chemical Company, Michigan, USA) [53] were added into the chamber (control group 1: nothing was added, control group 2: the same volume of PSS was added). With the start of phase three (P III), the PDMS chamber was stretched (15% radial stretch) while still continuously recording for one minute. Stretch was then again released to 0% stretch in phase four (P IV).

### Image processing

**Fig. 2:**
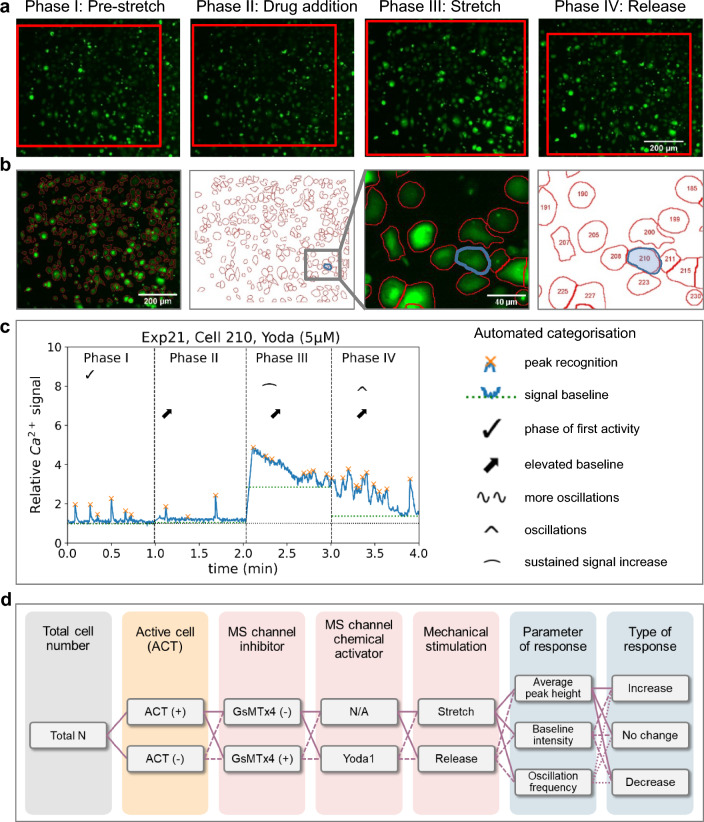
Stretch experiment in cardiac HL-1 cells followed by automated single-cell analysis of Ca^2+^ fluorescence. **a** Typical experiment in four phases P I to P IV (each 1 min long), P I: baseline at 1% pre-stretch, P II: addition of Yoda1 to the chamber, P III: chamber stretched by 15% for 1 min, P IV: baseline after release to 0% stretch (pre-stretch position). Slight chamber shifts during stretch experiments (red box) are corrected during image analysis. **b** Analysis of intracellular fluorescence levels based on automated segmentation of cells. Cell #210 in the image is tracked and highlighted for the stretch-effect on the circumference (shaded blue) **c** Relative Ca^2+^ fluorescence intensity in a representative single cell over the time-course of all four experimental phases. Automated detection of signal peaks (orange x’s) and baseline levels (green dotted lines) was implemented for quantitative analysis and subsequent automated categorisation of cellular response according to specified criteria (indicated by symbols). **d** Advanced study-design options and classification of data by initial cell status (orange), experimental conditions (pink) and cellular responses (blue)

Image processing and analysis were performed using ImageJ software [42] using a semi-automated macro. All fluorescence images were first corrected by subtraction of a dark image and a subsequent division by a flat-field correction image. During stretching/relaxation, it is inevitable that the section of the chamber which is within the FoV will shift to some extent (see Fig. 2a where the section showing the same cells is highlighted in red). Therefore, for cell segmentation, the time-lapse recording was split into individual stacks “Pre-stretch” – “Stretch” – “Release”. If an xy-drift appeared during different phases of the time-lapse recording, it was corrected for by using the ImageJ plugin “Linear Stack Alignment with SIFT” [29]. Since the "Stretch" phase (P III) has the best signal-to-noise ratio, we used it for segmentation: a threshold to define the cell areas was set manually, and the cells were segmented using the "Find maxima" operation which uses a Watershed algorithm (see Fig. 2b). After segmentation of the cells (Fig. 2b), the regions of interest (ROIs) representing the cells are transformed via the ImageJ plugin bUnwarpJ to match the position of the cells in the other stacks [2]. This allows for the registration of the same cell before, during and after stretch.

### Data processing

The visualisation and statistical analysis of the data received from image processing were further automated using the open-source programming language Python [23, 54]. To be able to validate every cell individually, their relative Ca^2+^ signal was calculated and plotted over time as the ratio of the signal intensity at time *t* (*S*_*t*_) and at the start of the experiment S_0_ (Fig. 2c). As most HL-1 cells already show spontaneous activity without mechanical stimulation, peaks in the signal were disregarded for the calculation of S_0_ (average of 3 s of the signal of any given experiment). The experiment was then divided into four phases as described above (each one minute long).

In the first phase (P I), the cells were examined either without any intervention (control) or being incubated with 5 µM GsMTx4 to presumably block subsequent activation of mechanosensitive, i.e. stretch-activated, channel activity. In the second phase (P II), Yoda1 was added to one group of samples, while nothing was added to control or GsMTx4 samples (the latter being labelled as ‘GsMTx4/–’; some control experiments were done by adding PSS instead of Yoda1, results are shown in the Supplementary Information, Suppl. Figure 1 and Suppl. Figure 2). Thus, the following experimental groups were present: (a) ‘–/–’ controls with no drug additions, (b) ‘GsMTx4/–’ with GsMTx4 present from P I but no Yoda1 in P II, (c) ‘–/Yoda1’ with no GsMTx4 in P I but Yoda1 added in P II and (d) ‘GsMTx4/Yoda1’ with GsMTx4 being present in P I and Yoda1 being added in P II. After a total of two minutes (P I + P II), 15% radial stretch was applied to the sample (third phase, P III), and after one more minute, the sample was relaxed back to 0% stretch (fourth phase, P IV). A peak recognition library (SciPy, *Find Peaks*) was used to identify peaks in the signal (indicated by orange x’s in Fig. 2c). To distinguish peaks from noise, the prominence value was set to 0.3 (prominence describing the vertical distance between the peak and lowest surrounding values).

From these data, the peak height as well as the baseline intensity were calculated for every phase of every individual cell. Next, the data were automatically categorised using a classification algorithm (as shown in Fig. 2d). First, the data from spontaneously active cells (defined by at least two peaks in the signal during P I) were separated from the data of cells activated by stretch only (in P III). ‘Cells activated by stretch’ were defined as showing less than two peaks in the first phase, but at least one peak in the third phase. As the signal may reach a state of sustained elevation after stretch (in P III), it is possible that no more than one peak can be detected, though there is a clear reaction to stretch. Details regarding sustained elevation are explained below. The phase of the first activity is indicated in the cells’ graphs by a checkmark (‘✓’ in Fig. 2c). Cells showing peaks but not fitting either category (random peaks), or showing no peaks at all were disregarded in the classification analyses. In phases II, III and IV, the baseline intensity was compared to the intensity in the previous phases I, II and III, respectively. An elevation in the baseline is indicated by an arrow ($\nearrow$) in Fig. 2c.

**Fig. 3:**
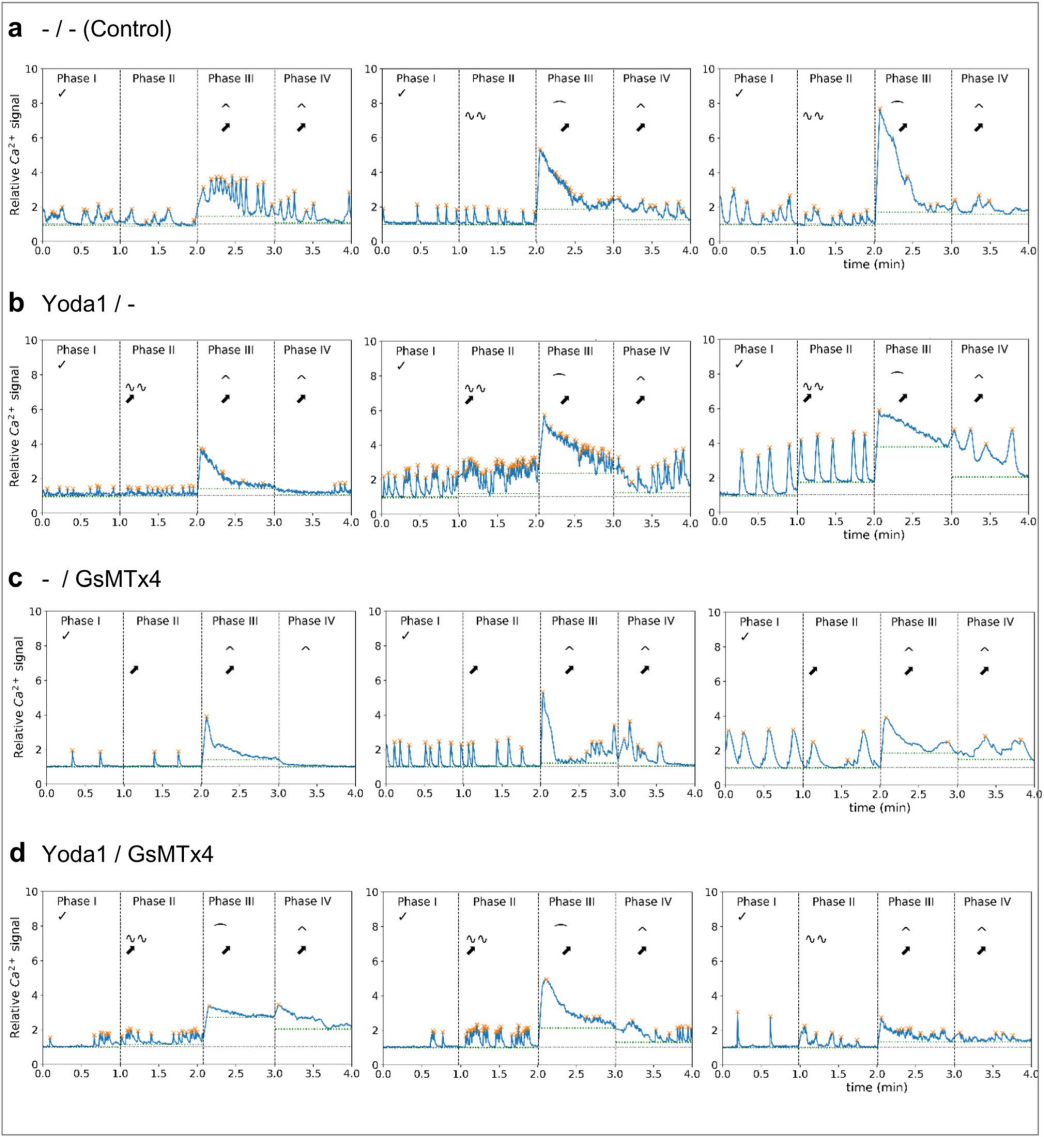
Single-cell Ca^2+^ levels in HL-1 cells in response to mechanical isotropic stretch activation and chemical triggers. A total of 12 experiments are shown with each plot tracking the relative Fluo-4 fluorescence signal intensity over time in the four phases of one experiment. Three experiments per condition are shown. Automated peak recognition and categorization of cellular responses was carried out as described in Fig. 2. **a** Control HL-1 cells without chemical modifiers, stretched in P III. **b** Cells treated with Yoda1 (5 µM) in P II, prior to stretch. **c** Cells incubated with GsMTx4 before P I and no addition of Yoda1 in P II. **d** Cells incubated with GsMTx4 before P I and addition of Yoda1 (5 µM) in P II, followed by mechanical stretch in P III. All selected cells were characterized as ‘active’ (ACT + , Fig. 2d), showing spontaneous Ca^2+^ oscillations in Phase I, and they also responded to stretch (increase/decrease, Fig. 2). Single-cell analysis allows a quantitative assessment and automated categorisation of individual cellular responses in hundreds of cells in parallel in a true high-content manner

The data were further examined for an increase in oscillations in P II (compared to P I), as a potential effect of Yoda1 sensitising Piezo1 channels, indicated in the graph by two tildes (~ ~). Additionally, the cells’ reaction to stretch and release in P III and P IV was further investigated. The algorithm checked whether the signal showed oscillations without a general increase in baseline intensity (compared to P II, indicated by a circumflex, ‘∧’), or a sustained increase in signal (indicated by a horizontal parenthesis, ‘ ∩ ’). To be recognised as a sustained signal increase, the baseline intensity of the respective phase’s first third had to be at least 1.5 units (of the relative signal) higher than in Phase II. If the baseline intensity stayed elevated by at least 20% of the initial elevation for the phase’s last two-thirds, the signal was categorised as sustained increase. Examples of single-cell data are shown in Fig. 3, split into the four experimental groups (control cells: ‘–/–’, cells treated with the mechanosensitive channel blocker GsMTx4: ‘-/GsMTx4 ‘, cells treated with Yoda1: ‘Yoda1/–’ and cells treated with GsMTx4 and Yoda1: ‘Yoda1/GsMTx4). The classification steps are shown in Fig. 2d.

Statistical analysis was performed and is listed in detail in Suppl. Table 1 and Suppl. Table 2. Two groups were compared at a time and first checked for normal distribution using the Shapiro–Wilk-Test. If either of them was not normally distributed, the Kruskal–Wallis *H* Test was applied. Otherwise, the One-Way ANOVA test was performed. *p* < 0.05 was considered statistically significant.

## Results

### High-content IsoStretcher technology for simultaneous investigation of stretch-activated ion channel function in a large number of cells

Cell stretcher technologies, such as the *IsoStretcher*, allow to examine mechanosensitive, i.e. stretch-activated, ion channels in their native cellular environment by stretching an elastomer chamber with adherent (or hydrogel-embedded) cells in culture. Figure 1e shows Fluo-4 labelled HL-1 cells visualised on a fluorescence microscope that ‘light up’ directly upon application of stretch. Before stretch (image on the left side), the cell is darker and difficult to distinguish from the background. After a stretch, it is larger in size and much brighter with a clear delineation to the background. The increase in cell size when applying stretch proves the successful transfer of stretch from the device onto the cell membrane area, the increase in fluorescence intensity indicates a higher Ca^2+^ level in the cell after stretch. This effect was reliably observed in all our experiments with HL-1 cells, in line with the general mechanosensitive properties of this cell type.

### High-content automated single-cell analysis of cellular Ca^2+^ signals in response to isotropic stretch

In cell stretch experiments on elastomer membranes, several hundreds of cells can be simultaneously monitored over time within the FoV (Fig. 2). To exploit that potential, we have developed an automated routine to evaluate these data on a single cell basis, as described in the Methods. Although cells inevitably move within that FoV to some (and often variable, depending on the radial location of the FoV) extent (red frame in Fig. 2a), it was possible to segment individual cells and to correctly align and scale these ROIs for each stretch experiment (Fig. 2b). For each ROI, the normalized fluorescence intensity was plotted over time, showing the time-course of relative cellular Ca^2+^ levels for individual cells (Fig. 2c). As the amount of Fluo-4 uptake can vary from cell to cell, it is important here to study relative changes in each individual cell (normalized to the baseline at start), which are independent of the dye concentration.

HL-1 cells exhibit spontaneous Ca^2+^ oscillations in the resting state [7]. Since in our experiments, HL-1 cell populations did not show a synchronized behaviour in both the non-stimulated and stimulated state (i.e. cells behaving independently and not as a functional syncytium), it was essential to evaluate Ca^2+^ imaging data on a single-cell basis because analysis of the integrated summation signal could have resulted in a loss of information about the oscillatory behaviour of the individual cells due to superposition. Figure 2c shows an exemplary Fluo-4 intensity curve for one cell over time from P I through P IV. The intensity curve was calculated for each individual ROI, and categorization was performed based on those single-cell intensity curves (Fig. 2d). Single-cell analysis enabled us to account for heterogeneous behaviour of the cells and more accurately statistically represent the changes in the cell behaviour in response to stretch and/or drug additions. Figure 3 shows a collage of three representative results from each of the four conditions for differential drug treatments (GsMTx4 and Yoda1 either both absent, present in isolation, or combined) for individual HL-1 cells during the phases P I through P IV, alongside their automated classification and analyses regarding the Ca^2+^ oscillation frequencies and Ca^2+^ levels in the different phases using the abbreviated classifiers introduced in the Methods. It can already be concluded from each of the treatment conditions (a)–(d) that there was substantial variability across individual cells of the same batch regarding oscillation frequencies and amplitudes as well as reaction to the same stretch level of 15% and subsequent relaxation, which inevitably justifies the approach of high-content analysis from a substantial number of single cells for each condition for sub-population identification. Consistently, however, stretch proved to induce a sort of ‘gain-of-function’ reaction of the Ca^2+^ signals, with a sustained Ca^2+^ transient response and/or an increase in the Ca^2+^ oscillation frequency, regardless of drug treatment, and a subsequent resolution thereof during the stretch relaxation phase P IV.

### Quantitative classification of HL-1 cellular responses to stretch and GsMTx4/Yoda1 treatment

**Fig. 4:**
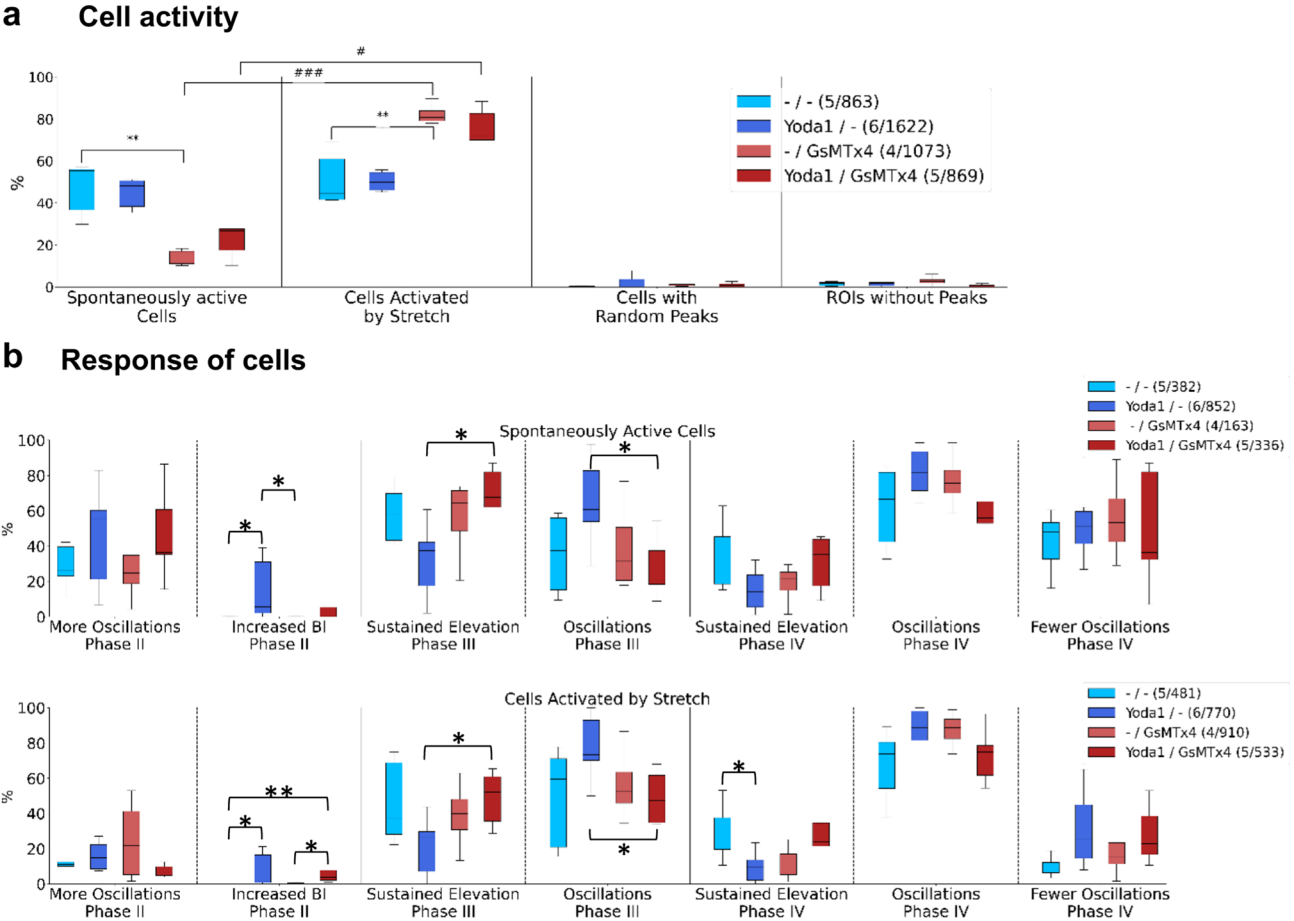
Characterization of cellular activity and cellular response. **a** Automated categorization of HL-1 cell activity in four groups: ‘Spontaneously active cells’ exhibit at least two Ca^2+^ peaks within Phase I (P I, pre-stretch), ‘Cells activated by stretch’ only show peaks after mechanical stimulation in Phase III (P III, stretch), ‘Cells with random peaks’ show some activity, but cannot be classified as either of the two first groups, ‘ROIs without peaks’ do not show any activity at all (most probably reflecting dead cells or misplaced ROI). Percentages were assessed separately for each sample and summarized in boxplots (box: quartiles, whiskers: 5–95%). Statistical analysis shows that cells pre-incubated with GsMTx4 before P I (no Yoda1 treatment in P II) show significantly less spontaneous activity compared to control cells (**p < 0.01). On the contrary, they are significantly more likely to be first activated (show first peaks) by stretch in P III (**p < 0.01). More data regarding the reaction to stretch of both groups are displayed in Fig. 5. **b** The cells’ response to the previously described criteria (see Fig. 2) was plotted as boxplots. Furthermore, the data have been separated into two graphs, showing data for spontaneously active cells (top) and cells activated by stretch only in P III (bottom), which may represent different physiological states. (m/n) states the number of individual cells n from m replicates

During analysis of the data gained from the experiments, it became clear that there were two types of cell activity. The first activity type includes cells showing spontaneous activity already before any stimulus was applied. This activity manifested itself as discernible oscillating peaks in the fluorescence traces of P I and/or P II. Cells of the second activity type did not show any spontaneous activity there but then reacted to mechanical stimulation in P III (cells of the spontaneous type also were responsive in P III, see Fig. 2d). Figure 4a shows the distribution of cells across these two groups. Cells that were not treated with GsMTx4 were equally distributed among the groups, independently of the treatment with Yoda1. The treatment with GsMTx4 markedly changed this behaviour. In the presence of GsMTx4, only about 15% of cells showed spontaneous activity before stretch (about 20% after Yoda1 treatment, not significant), and 70–80% were only active after stimulation by stretch, which is significantly more than in the absence of GsMTx4. The remaining cells showed either random peaks (less than two peaks in P I) or no peaks at all, however, at a low proportion (< 5%, Fig. 4a). Statistical analysis shows a highly significant increase in cellular Ca^2+^ oscillation activity caused by treatment with GsMTx4 in cells only activated by stretch (one way ANOVA, *p* < 0.01) while in spontaneously active cells, GsMTx4 had the significantly opposite effect.

For subsequent further in-depth analysis of Ca^2+^ signal responses, cells were separated into the two major types of presented activity, i.e. spontaneously active cells vs. cells only activated by stretch. This was done to consider that HL-1 cells were not synchronised here, and analysis, therefore, could be tuned to those two cell types. This information would have likely been lost in a summation signal approach. Figure 4b shows the categorisation of the cells’ responses. First, the effect of Yoda1 addition in P II was examined by comparing the number of oscillations in P I and P II. When looking at spontaneously active cells, Yoda1 had a tendency to increase the number of peaks per minute (in P II and P III, not significant), independently of the GsMTx4 treatment which seemingly did not have much effect. Cells without spontaneous activity (only activated by stretch), overall, showed a similar trend here in P III upon stretch, but to a much lesser extent in P II. In contrast, Yoda1 had a much more pronounced effect on baseline intensity (BI), significantly increasing the latter in P II across the activity types, an effect that was almost blunted in the presence of GsMTx4 (Fig. 4b, second panels).

When examining the cells’ behaviour during the stretch, two predominant reactions were observed. The first case shows a sustained elevation of the signal or oscillations throughout P III at stable baseline intensity. Sustained elevation is characterised by an initial peak in the signal, usually with a higher amplitude than previous peaks and a continuously increased BI compared to P I and P II (with or without further oscillations) (also see examples in Fig. 3). In the second case, the signal is oscillating, but no increase in BI is recognised. The two cell activity types show very similar results arguing in favour that stretch response was universal regardless of initial cell activity. Compared to control cells (‘–/–’), cells treated with only Yoda1 are less likely to reach sustained elevation and are more likely to keep oscillating (no significant differences). Cells treated with only GsMTx4 behave similarly to control cells. When additionally treated with Yoda1, sustained elevation is slightly more prevalent compared to controls and accordingly, the prevalence of oscillations is decreased (significant difference between ‘Yoda1/–’ and ‘Yoda1/GsMTx4’ cells).

The same examinations were done for P IV, after the stretch was released. The results are similar to P III, with the exception that the behaviour of GsMTx4-treated cells is closer to cells treated with only Yoda1 than to control cells (significant difference between ‘–/–’ and ‘Yoda1/–’ cells activated by stretch only). In addition, the number of oscillations in P IV was compared to P II. For spontaneously active cells, in every group, about 50% of the cells show fewer oscillations in P IV than in P II. Looking at cells activated by stretch, very few control cells and cells treated with GsMTx4 showed fewer oscillations in P IV than in P II, but when treating cells with Yoda1 (and GsMTx4 + Yoda1), about 30% of cells exhibited a decrease in oscillations in P IV.

**Fig. 5:**
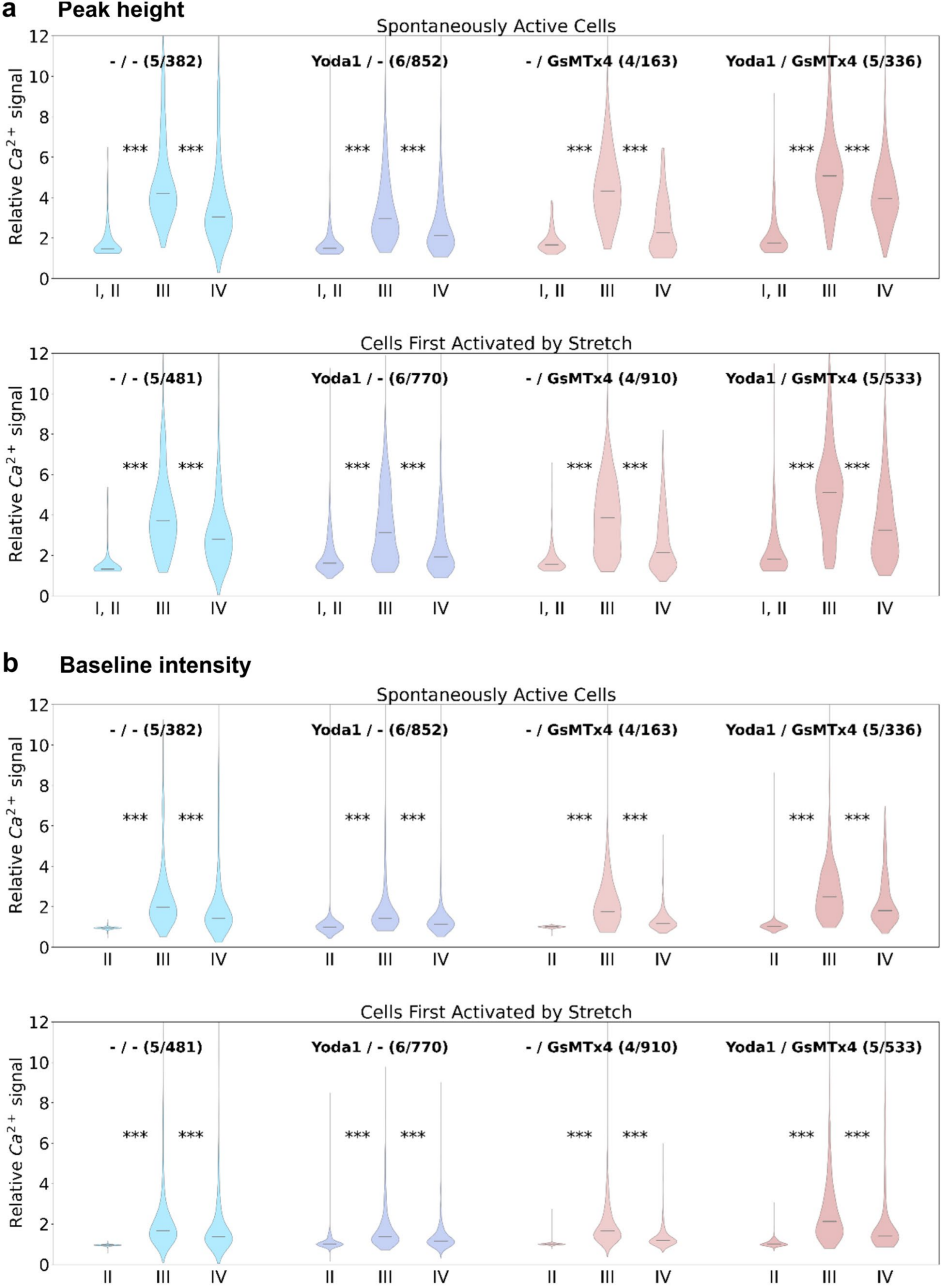
Quantitative assessment of cellular Ca^2+^ levels in different experimental conditions. **a** Violin plots show the peak height of the Ca^2+^-dependent fluorescence signal intensity through Phases I to IV of the experiment. In Phases I and II, the average peak height is plotted. In Phases III and IV, only the highest peak of the initial response to stretch (first 20 s) and release was considered. **b** Violin plots show the increase in baseline intensity through stretch (P I is used as a reference and, therefore, disregarded in this plot). Comparing each phase to the following, stretch evidently has a significant impact on the peak height as well as the baseline intensity (***p < 0.001)

The results of the Ca^2+^ peak height analysis clearly support the hypothesis of stretch-induced calcium influx. From the violin plots in Fig. 5a, the signal in P III (compared to P I, P II) is elevated for every scenario, independent of cell activity type and treatment (all Kruskal–Wallis H Test, *p* < 0.01). Furthermore, when stretch is released in P IV, the signal consistently decreases (though not returning back to peak height levels before stretch). Independent of cell activity type, the peak height in cells treated with Yoda1 appears to be smaller than in control cells. Cells treated with GsMTx4 behave similarly to control cells (no significant difference), and the highest peaks were observed in cells treated with both GsMTx4 and Yoda1.

Finally, the BI's development throughout the experiments was examined (Fig. 5b). As for the peak height, the baseline is generally elevated by stretch and decreases during release, though even after release, the BI still remained higher than during P I and P II. Consistent with the results for peak height, BI was overall the lowest in cells treated with Yoda1, a little higher in control cells and cells treated with GsMTx4, and the highest in cells treated with GsMTx4 and Yoda1.

## Discussion

Cardiac mechano-electric coupling has been known and systematically studied since the early years of the twentieth century [37]. However, mechanosensitive (stretch-activated) ion channels have only been considered as possible transducers of mechanical stimuli in cardiac cells after their discovery in chick-skeletal muscle [19] and follow-up studies in cardiomyocytes [9, 10]. Among the known mechanosensitive ion channels, the TRP-type channels TRPC1, TRPC3 and TRPC6 were in most cases experimentally established to adequately fulfil the role of cardiac mechanosensors [56], until direct activation of the mammalian TRP ion channels by membrane stretch in patch clamp experiments has become controversial [18, 32]. In addition to the ample evidence for a central role Piezo1 channels play in vascular (patho)physiology [28, 38, 40], they were recently also shown to function as the primary Ca^2+^ permeable mechanoreceptors directly activated by membrane stretch in cardiac cells [27, 59].

The contribution of Piezo1 mechanosensitive ion channels in cardiac pathophysiology has recently been reported. The examples include cardiac hypertrophy [27, 59], and arrhythmia [35, 41]. This is not surprising given that mechanosensitive ion channels have long been postulated to play a major role in mechano-electric feedback, which involves changes in mechanical load (stress, strain) in the cardiac wall and blood vessels [37]. The exact mechanism of how mechanosensitive channels contribute to coupling mechanical and electrical stimuli in cardiac tissue is still unknown. Such open questions create a need for new technologies with higher throughput than classical electrophysiology to more efficiently tackle these research tasks.

In our study, we have established a method to investigate mechanotransduction with isotropic planar cell stretcher technology and analyse the cellular response to stretch on a single-cell basis. The combination of the *IsoStretcher* system, Ca^2+^ fluorescence, and automated analysis routines allows for such investigations at a high-content level applied to a multitude of single cells simultaneously. This is particularly important in view of not only being able to secure data from a large number of individual cells but also to include all of the data entries in a classification grouping approach to identify subpopulations of biological responders as well as their relative fractions. It has been well known for some time now that so-called ‘observer-bias’ represents one of the most compromising variables in providing robust and reproducible results in biomedical research [5, 33]. It has even been claimed that many discoveries in basic and preclinical research may not stand the test of time [5, 33] due to poor reproducibility. Among many reasons, observer bias in selectively choosing ‘good’ cells and discarding ‘bad’ cell responses (according to underlying hypothesis expectations) from entering data analysis represents a major influence, particularly when involving tedious single-cell experiments typically yielding a low number of observations. Thus, our high-content *IsoStretcher* approach falls in line with current recommendations and strategies to reduce observer bias [6, 31].

### Application of the high-content stretch analysis of Ca^2+^ signaling in cardiac HL-1 cells

In this study, we investigated the role of Piezo1 ion channels in HL-1 cardiac cells using Ca^2+^ fluorescence imaging combined with the *IsoStretcher*, which enabled high-content studies of the effect of membrane stretch on large populations of these cells. Although HL-1 cells are derived from a mouse atrial cardiomyocyte tumour lineage [7], they show a differentiated cardiac phenotype with spontaneous contractions [58]. HL-1 cells are often used as a substitute for adult cardiomyocytes, in particular in functional screening studies [57] or in tissue engineering [16], due to their relatively easier handling over adult primary cardiomyocytes and their adherent behaviour [61]. This adherent behaviour on silico-elastomer substrates renders them much more feasible for use in PDMS-substrate stretch experiments [43] over adult ventricular cardiomyocytes, which will not adhere to such substrates well and would have to be embedded in hydrogels of tuneable stiffness [13, 14]. This rather tedious process, for adult primary cardiomyocytes, however, is necessary, as isolated load-free adult cardiomyocytes show markedly different Ca^2+^ signalling as opposed to hydrogel-embedded cardiomyocytes that are under mechanical load [26]. Thus, to establish our high-content quantitative stretch-Ca^2+^ signalling platform in 2D adherent cardiac cells as a step before turning to more elaborate ‘cell-in-a-gel’ adult cardiomyocyte settings, we chose the use of HL-1 cells here. Apart from Piezo1 [12], HL-1 cells have also been shown to express mechanosensitive TRPM4 [24] and TRPC3 [36] channels. However, TRP channels have recently been reported to be insensitive to direct membrane stretch activation [32], thus, our *IsoStretcher* setting is mainly tracking Piezo1-mediated Ca^2+^ fluctuations. Very recently, we investigated the functional coupling of Piezo1 and TRPM4 channels in HL-1 cells [20], showing that Yoda1 is activating Ca^2+^ influx in HL-1 cells through Piezo1 channels, confirming our findings in the present study.

Our results show a significant increase in global fluorescence in most HL-1 cells within the FoV that was analysed, indicating a large influx of Ca^2+^ induced by stretching the cells. To document the involvement of Piezo1 in these experiments, we employed Yoda1, a specific Piezo1 channel agonist [53]. The addition of micromolar concentrations of Yoda1 before the application of stretch resulted in an increase in Ca^2+^ fluorescence confirming the activation of the Piezo1 channels as the conduits for extracellular Ca^2+^ (see Fig. 4b, oscillations and BI in P II). As temperature can have an impact both on oscillations and on mechanosensing, it should be noted that all experiments were equally carried out at room temperature.

The two activity phenotypes, i.e. with and without spontaneous activity, possibly reflected different physiological states of cells identified in our experiments. Both were activated by the addition of Yoda1 as well as by mechanical stimulation using the *IsoStretcher* system. The fluorescence signal often reached sustained elevation without further fluorescence peaks (see Fig. 4b) suggesting the formation of a steady state between Ca^2+^ entry and extrusion mechanisms in the stretched cells compared to control cells [55]. In contrast, however, the signal of most cells treated with Yoda1 continued to oscillate, albeit stronger than before stretch (see Fig. 4b). The here executed experiments cannot unambiguously explain the effect of seemingly smaller reaction to stretch under the influence of Yoda1 (see Fig. 5a, b).

In addition to the Piezo1 agonist Yoda1, we also used the generic mechanosensitive channel blocker GsMTx4 [50] to examine the Piezo1 channel contribution to intensity changes in cell fluorescence. Results obtained with the GsMTx4 peptide were overall consistent with the reported effect of GsMTx4 on Piezo1 channels. As previously shown in patch clamp studies [3], extracellularly added micromolar concentrations of GsMTx4 reversibly inhibited ∼80% of the Piezo1 current in outside-out HEK293 cell patches. Importantly, GsMTx4 was active on closed channels. Consistent with these patch-clamp results, the addition of GsMTx4 in our study did not result in the reduction of Ca^2+^ fluorescence intensity. On the contrary, when looking at the peak amplitude and baseline intensity signals, HL-1 cells treated with Yoda1 and GsMTx4 consistently showed the strongest signal throughout all phases indicating that GsMTx4 was largely ineffective in blocking Piezo1 channels activated by Yoda1 in our experiments. And even cells only treated with GsMTx4 showed stronger signals than controls or cells treated with Yoda1 alone. This lack of the expected GsMTx4 effect possibly stems from its amphipathic properties, given that GsMTx4 interacts with the lipid bilayer acting as a ‘‘mobile reserve’’ of membrane material [17]. Its penetration into the membrane bilayer shifts between shallow and deep penetration depending on bilayer tension, which distorts the distribution of membrane tension and thus, to a different extent, affects the transfer of force from the bilayer to an inherently stretch-activated ion channel, like Piezo1 [8, 52]. It is, therefore, likely that the lack of Piezo1 channel blockage by GsMTx4 in our experiments was in addition a consequence of the variable GsMTx4 penetration into the cell membrane during stretching of HL-1 cells by the *IsoStretcher* device.

Importantly, in cardiomyocytes, including HL-1 cells, the expression of mechanosensitive channels has been shown for Piezo1 [21], and various TRP-type ion channels [60]. However, of these channels, only Piezo1 can be activated by membrane stretch [32]. Therefore, in our *IsoStretcher* configuration, we are confident that our results reflect the activation of Piezo1 channels. Cell activity was, nevertheless, somewhat influenced by treatment with GsMTx4 because only about 10–20% of cells treated with GsMTx4 (or GsMTx4 and Yoda1) were spontaneously active, whereas 70–80% were first activated through the application of stretch. This difference is statistically significant between the activity groups, as well as when compared with control and GsMTx4 treatment (see Fig. 4a). We believe that these results are consistent with the variability of the GsMTx-4 membrane penetration during stretch since opening of the Piezo1 channels increases the local membrane area around the channel, thus increasing the probability of this spider peptide to insert deeper into the expanded membrane and, therefore, block the channels more efficiently [17].

## Conclusions

In summary, our study introduces a method for high-content analysis of cellular responses to mechanical stimuli following isotropic stretch. The advantages of such an approach include (i) the adaptability of the stretch devices to simultaneous microscopic observations and measurements of fluorescence signals in a larger population of cells, (ii) acquisition of statistically meaningful data in a population of cells irrespective of their heterogeneity due to the cell growth phase, protein expression, their level of confluence, or other disparity in physiological states, and (iii) easily operated application of pharmacological agents for identification and characterization of stretch-activated membrane proteins participating in cellular processes under study. Our method is also applicable in future experiments using synchronous cell populations for the analysis of the Piezo1 channel function at specific stages of the cell cycle, or in studies of fully differentiated cardiomyocytes embedded in hydrogels under load. The use of the *IsoStretcher* system in this study enabled examination of the presence of Piezo1 stretch-activated channels in HL-1 cardiac cells as contributors to intracellular Ca^2+^ increase in these cells upon stretching their membrane. Together with the recent studies documenting the function of Piezo1 ion channels as primary mechanoreceptors in cardiac ventricular myocytes [27, 59], our study suggests a similar role for Piezo1 in atrial HL-1 cardiomyocytes indicating the presence of these stretch-activated channels throughout the heart tissue.

### Supplementary Information

Below is the link to the electronic supplementary material.

Supplementary file1 (DOCX 1016 KB)

## Data Availability

The datasets generated and analysed during the current study are available from the corresponding author upon request. Data are located in controlled access data storage at Friedrich-Alexander-Universität Erlangen-Nürnberg (FAU).
