# Supplementary material for: High-content method for mechanosignaling studies using IsoStretcher technology and quantitative Ca2+ imaging applied to Piezo1 in cardiac HL-1 cells
Source: Cell Mol Life Sci. 2024 Mar 14;81(1):140. doi: 10.1007/s00018-024-05159-6 (PMC10940437; doi:10.1007/s00018-024-05159-6)
Supplement: Supplementary file 1 — Supplementary file1 (DOCX 1016 KB) [file 18_2024_5159_MOESM1_ESM.docx]

# Supplementary Material

**Suppl. Table 1:** Peak height of Ca^2+^ signal compared pairwise between groups via one-way ANOVA or Kruskal-Wallis: n.s., not significant; *, p < 0.05; **, p < 0.01; ***, p < 0.001. Upper-right half shows results for spontaneously active cells, lower-left half shows results for cells first activated by stretch.

| spontaneously  active cells  cells activated  by stretch | | | **Control** | | | | | | **GsMTx4** | | | | | |
| --- | --- | --- | --- | --- | --- | --- | --- | --- | --- | --- | --- | --- | --- | --- |
|  |  |  | **-** | | | **Yoda1** | | | **-** | | | **Yoda1** | | |
|  |  |  | **PI+II** | **PIII** | **PIV** | **PI+II** | **PIII** | **PIV** | **PI+II** | **PIII** | **PIV** | **PI+II** | **PIII** | **PIV** |
| **Control** | **-** | **PI+II** |  | *** |  | ns |  |  | *** |  |  | *** |  |  |
|  |  | **PIII** | *** |  | *** |  | *** |  |  | ns |  |  | *** |  |
|  |  | **PIV** |  | *** |  |  |  | *** |  |  | *** |  |  | *** |
|  | **Yoda1** | **PI+II** | *** |  |  |  | *** |  |  |  |  | *** |  |  |
|  |  | **PIII** |  | *** |  | *** |  | *** |  |  |  |  | *** |  |
|  |  | **PIV** |  |  | *** |  | *** |  |  |  |  |  |  | *** |
| **GsMTx4** | **-** | **PI+II** | *** |  |  |  |  |  |  | *** |  | ** |  |  |
|  |  | **PIII** |  | ns |  |  |  |  | *** |  | *** |  | *** |  |
|  |  | **PIV** |  |  | *** |  |  |  |  | *** |  |  |  | *** |
|  | **Yoda1** | **PI+II** | *** |  |  | *** |  |  | *** |  |  |  | *** |  |
|  |  | **PIII** |  | *** |  |  | *** |  |  | *** |  | *** |  | *** |
|  |  | **PIV** |  |  | *** |  |  | *** |  |  | *** |  |  |  |

**Suppl. Table 2:** Baseline Intensity of Ca^2+^ signal compared pairwise between groups via one-way ANOVA or Kruskal–Wallis: n.s., not significant; *, p < 0.05; **, p < 0.01; ***, p < 0.001. Upper-right half shows results for spontaneously active cells, lower-left half shows results for cells first activated by stretch.

| spontaneously  active cells  cells activated  by stretch | | | **Control** | | | | | | **GsMTx4** | | | | | |
| --- | --- | --- | --- | --- | --- | --- | --- | --- | --- | --- | --- | --- | --- | --- |
|  |  |  | **-** | | | **Yoda1** | | | **-** | | | **Yoda1** | | |
|  |  |  | **PII** | **PIII** | **PIV** | **PII** | **PIII** | **PIV** | **PII** | **PIII** | **PIV** | **PII** | **PIII** | **PIV** |
| **Control** | **-** | **PII** |  | *** |  |  |  |  |  |  |  | *** |  |  |
|  |  | **PIII** | *** |  | *** |  | *** |  |  | ns |  |  | *** |  |
|  |  | **PIV** |  | *** |  |  |  | *** |  |  | *** |  |  | *** |
|  | **Yoda1** | **PII** |  |  |  |  | *** |  |  |  |  |  |  |  |
|  |  | **PIII** |  | *** |  | *** |  | *** |  |  |  |  | *** |  |
|  |  | **PIV** |  |  | *** |  | *** |  |  |  |  |  |  | *** |
| **GsMTx4** | **-** | **PII** |  |  |  |  |  |  |  | *** |  |  |  |  |
|  |  | **PIII** |  | ns |  |  |  |  | *** |  | *** |  | *** |  |
|  |  | **PIV** |  |  | *** |  |  |  |  | *** |  |  |  | *** |
|  | **Yoda1** | **PII** | *** |  |  |  |  |  |  |  |  |  | *** |  |
|  |  | **PIII** |  | *** |  |  | *** |  |  | *** |  | *** |  | *** |
|  |  | **PIV** |  |  | *** |  |  | *** |  |  | *** |  | *** |  |

# Supplemental Figures


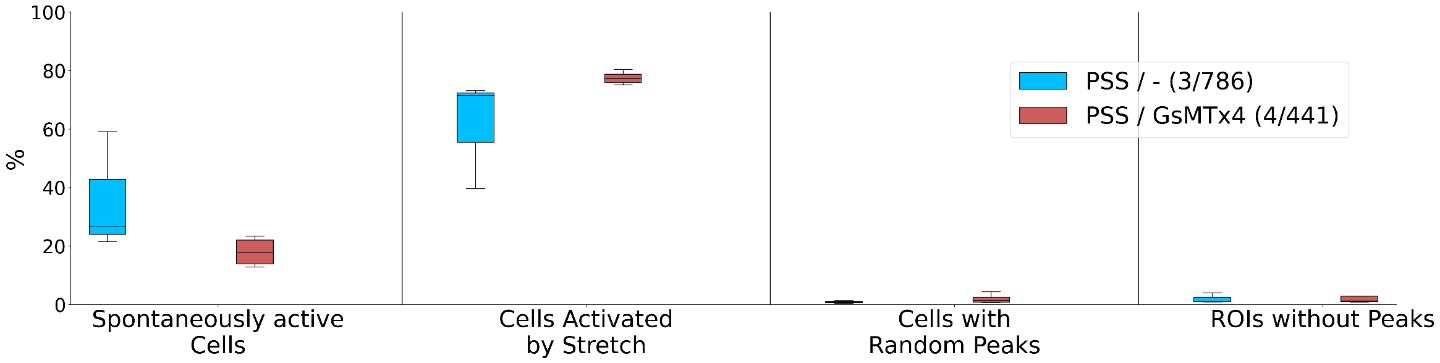

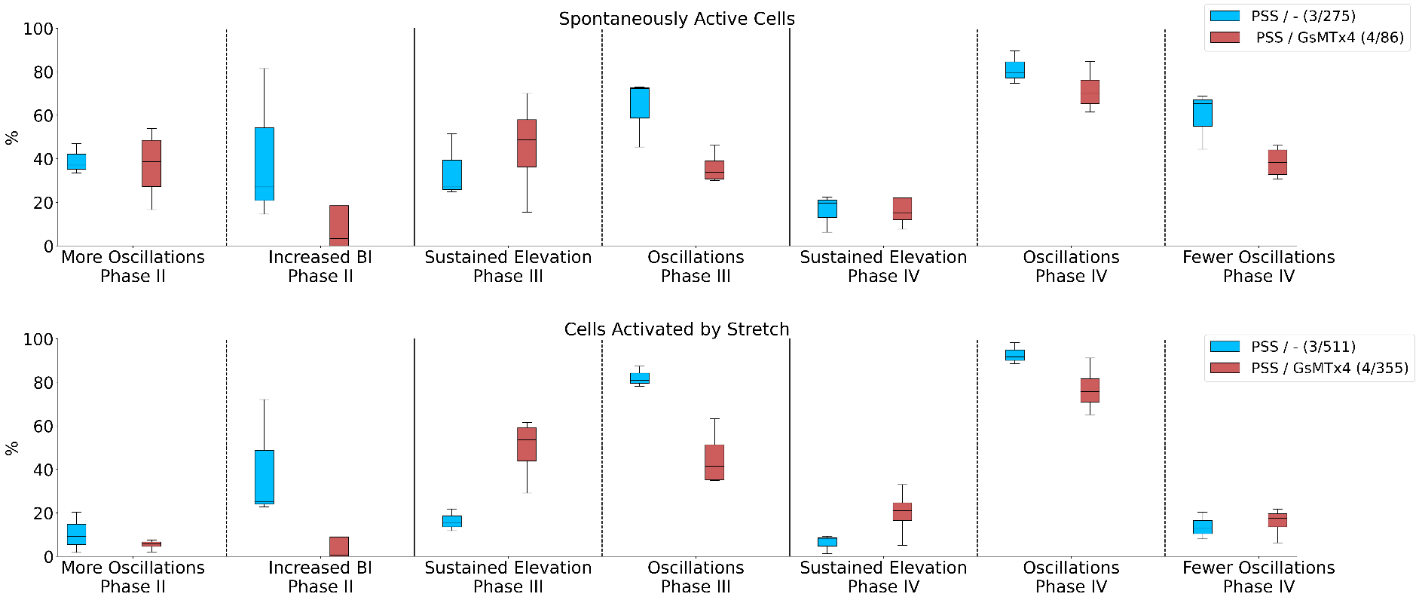


**a**

**b**

**Cell activity**

**Response of cells**

**Suppl. Fig. 1** Characterization of cellular activity and cellular response (addition of PSS in Phase II). (a) Automated categorization of HL-1 cell activity in four groups: ‘spontaneously active cells’ exhibit at least two Ca^2+^ peaks within Phase I (pre-stretch), ‘cells activated by stretch’ only show peaks after mechanical stimulation in Phase III (stretch), ‘cells with random peaks’ show some activity, but cannot be classified as falling into either of the two first groups, ‘ROIs without peaks’ do not show any activity at all (most probably reflecting dead cells or misplaced ROI). Percentages were assessed separately for each sample and are summarised in boxplots (box: quartiles, whiskers: 5-95%). Cells pre-incubated with GsMTx4 show less spontaneous activity compared with control cells. They are more likely to be first activated (show first peaks) by stretch. More data regarding the reaction to the stretch of both groups is displayed in Suppl. Fig. 2. (b) The cells’ response to the previously described criteria (see Fig. 2) was plotted as boxplots. Furthermore, the data have been separated into two graphs, showing data for spontaneously active cells (top) and cells activated by stretch (bottom), which may represent different physiological states.


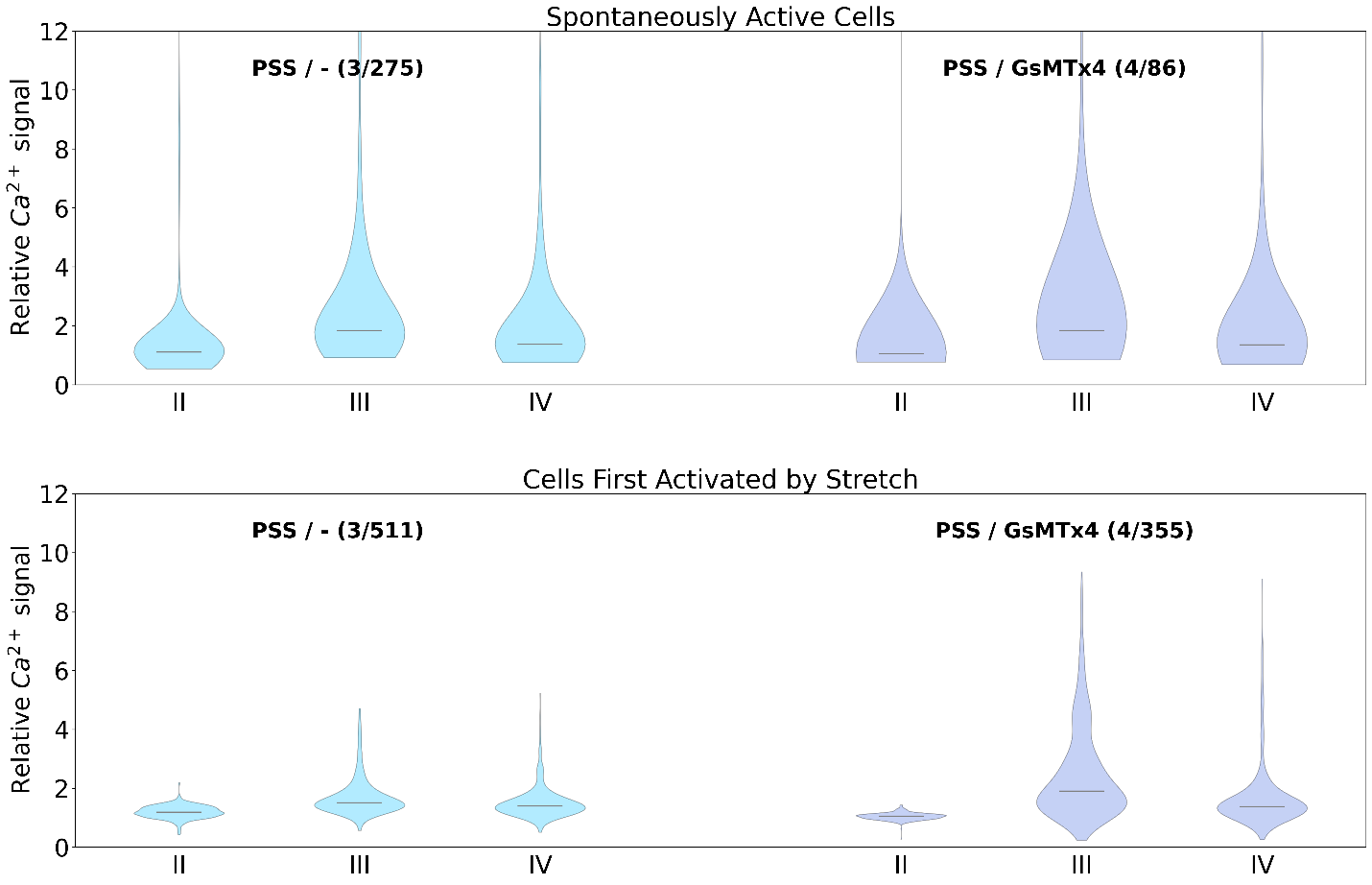

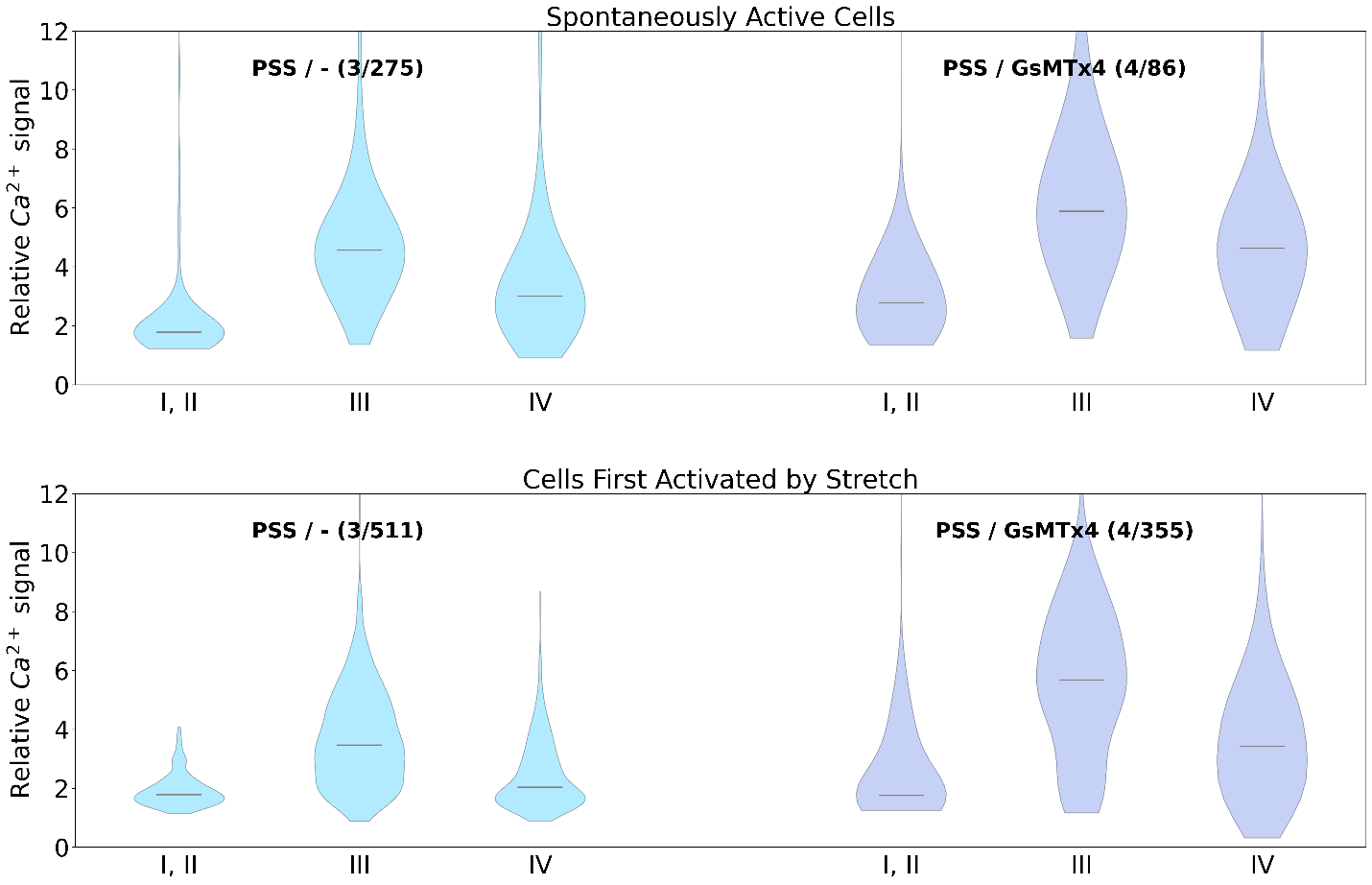


**b**

**Peak height**

**Baseline intensity**

**a**

**Suppl. Fig. 2** Quantitative assessment of cellular Ca^2+^ levels in cells with and without GsMTx4 treatment (addition of PSS in Phase II). (a) Violin plots show the peak height of the Ca^2+^ dependent fluorescence signal intensity through Phases I to IV of the experiment. In P I and P II, the average peak height is plotted. In P III and P IV, only the highest peak of the initial response to stretch (first 20 s) and release was considered. (b) Violin plots show the increase in baseline intensity through stretch (P I is used as a reference and therefore disregarded in this plot). Comparing each phase to the following, stretch evidently has an impact on the peak height as well as the baseline intensity.
